# Critical Care Management of Patients with COVID-19: Early Experience in Thailand

**DOI:** 10.4269/ajtmh.20-0442

**Published:** 2020-05-18

**Authors:** Ranistha Ratanarat, Chaisith Sivakorn, Tanuwong Viarasilpa, Marcus J. Schultz

**Affiliations:** 1 Division of Critical Care, Department of Medicine, Siriraj Hospital, Mahidol University, Bangkok, Thailand;; 2 Department of Clinical Tropical Medicine, Mahidol University, Bangkok, Thailand;; 3 Mahidol–Oxford Tropical Medicine Research Unit (MORU), Mahidol University, Bangkok, Thailand;; 4 Nuffield Department of Medicine, University of Oxford, Oxford, United Kingdom;; 5 Department of Intensive Care, Amsterdam University Medical Centers, location ‘AMC’, Amsterdam, The Netherlands

## Abstract

Since late December 2019, the world has been challenged with an outbreak of COVID-19. In Thailand, an upper middle–income country with a limited healthcare infrastructure and restricted human resources, nearly 3,000 confirmed COVID-19 cases have been reported as of early May 2020. Public health policies aimed at preventing new COVID-19 cases were very effective in halting the pandemic in Thailand. Case fatality in Thailand has been low (1.7%), at least in part due to early stratification according to risk of disease severity and timely initiation of supportive care with affordable measures. We present our initial experience with COVID-19 in Thailand, focusing on several aspects that may have played a crucial role in curtailment of the pandemic, and elements of care for severely ill COVID-19 patients, including stratification, isolation, and affordable diagnostic approaches and supportive care measures. We also discuss local considerations concerning some proposed experimental treatments.

## INTRODUCTION

COVID-19, a respiratory infection that originated in Wuhan, China, in late 2019, by now has spread to almost all countries worldwide.^1^ The numbers of cases are still rising in most countries, at an unprecedented and worrisome speed.^2^ Thailand, an upper middle–income country in Southeast Asia, was one of the first countries outside of China to report COVID-19 cases. The first case was a traveler from Wuhan, China,^3,4^ followed by local transmission to a public transport driver in Bangkok.^5^ Since then, nearly 3,000 confirmed COVID-19 cases have been reported in Thailand.^2^

Among several problematic aspects of COVID-19, its transmissibility may be most challenging. Infected individuals produce large quantities of virus in their upper airways in the prodromal phase, during which they remain mobile and able to continue their usual activities, and thus able to transmit the disease to many contacts. This is very different from the earlier SARS, in which viral shedding occurred mainly when patients manifested severe illness.^6^ The higher transmissibility of COVID-19 makes the outbreak much more difficult to control, thus increasing the size of the pandemic. As of early May, however, Thailand’s response to COVID-19 seems successful. Public health measures,^7,8^ together with assistance from village health volunteers and the general public, limited the number of COVID-19 cases, and the pandemic is by now, for the most part, halted in Thailand.^9^

Since soon after the start of the outbreak, a tremendous volume of reports on clinical management of COVID-19 has been published, mainly from and for high-income countries. Some of the suggestions and recommendations in those reports are rather unsuitable for middle-income countries, where resources are usually severely limited, as in Thailand. The objective of this report is to outline our initial experience with the management of COVID-19 patients requiring intensive care unit (ICU) admission. After highlighting the most important measures taken in Thailand, we discuss affordable diagnostic approaches and supportive care measures taken in Thai hospitals. We also discuss local considerations regarding experimental treatments.

## PUBLIC HEALTH AND LOCAL MEASURES AGAINST THE COVID-19 PANDEMIC

In Thailand, as in Wuhan, China,^10^ several measures may have limited the progression of COVID-19 (Table 1), possibly reducing the need for hospitalization and eventually ICU admission for ventilatory support.

**Table 1:** Public health and general measures against COVID-19 in Thailand

Measure	Purpose/aim
Case definition	
* *Frequent updates of definition of PUIs	Earlier identification and isolation of cases
* *Laboratory screening and isolation	
* *Maximizing COVID-19 PCR testing and intensive active surveillance	Earlier identification of cases
* *Completion of case isolation including every probable/confirmed COVID-19 case or PUIs with negative initial PCR testing results	May limit transmission
* *Limited traveling and social contacts	May limit transmission
Lung imaging	
* *Use lung ultrasound next to chest radiography and chest computed tomography	Earlier diagnosis of pneumonia may allow earlier isolation and start of treatment
Antiviral treatment before intensive care unit admission	
* *Favipiravir for all COVID-19 patients with pneumonia or hypoxemia	May prevent deterioration and may reduce the need for intensive care unit admission and escalation of respiratory support

PUIs = patients under investigation.

### Case definition.

In Thailand, the definition of “patients under investigation” (PUIs) was updated frequently, to improve early identification of COVID-19 cases. The current definition includes 1) a history of fever or documented temperature ≥ 37.5°C and/or any respiratory symptom accompanied by a history of high risk for COVID-19 infection in the 14 days before the onset of symptoms, 2) cases of suspected pneumonia with unknown etiology, 3) clusters of sick individuals, and 4) suspected cases among healthcare workers.^8^

### Laboratory screening and isolation.

In Thailand, the diagnosis of COVID-19 is confirmed by reverse transcriptase–PCR (RT-PCR). In critically ill patients, sputum or tracheal suction specimens are preferred over nasopharyngeal or throat swabs for testing because the lower respiratory tract samples are most often testing positive for the virus.^11^ It is recommended that every confirmed case, with or without symptoms, is hospitalized or kept under observation at a designated isolation facility. After the clinical condition has improved, and if no complications are observed, cases are considered for transfer to a designated hospital or temporary patient ward for COVID-19, continuing isolation for at least 14 days from the date of onset. For asymptomatic infection, which is usually identified by contact tracing, the isolation duration will be counted from the day of diagnosis. After that, it is recommended that, for an additional 2 weeks, patients 1) wear a surgical mask at all times and pay extra attention to respiratory hygiene, such as coughing or sneezing in a flexed elbow or tissue, and disposing of used tissue into a closed bin; 2) avoid close contact with vulnerable populations; and 3) seek medical care immediately whenever respiratory symptoms recur or worsen.

### Lung imaging.

A chest radiograph (CXR) in patients with moderate COVID-19 may show nonspecific multi-lobar opacities that rapidly progress over the first days of illness. Chest computed tomography (CT) findings in COVID-19 patients are more specific, showing bilateral, multi-lobar “ground-glass opacification” with the so-called crazy paving.^12^ Because of lack of CT scanners in most Thai hospitals, CXR remains the first imaging modality for diagnosis. It should be noted that both “ground-glass opacification” and “crazy paving” cannot be seen on a CXR, and also that a CXR may not show abnormalities in the early stages of this disease.^13^

Lung ultrasound (LUS) is an attractive alternative for screening and monitoring COVID-19.^14^ In our experience, LUS is able to detect COVID-19 earlier than a CXR, and daily LUS combined with physical examination facilitates early detection of progression of the disease. However, LUS requires specific training, which is a limitation in settings where ultrasound is not yet as extensively used as in Thai hospitals. We also have promising experience with the “Kigali modification of the Berlin definition for acute respiratory distress syndrome (ARDS)” in the early detection and management of COVID-19 patients.^15^ The Kigali modification allows the use of pulse oximetry instead of blood gas analysis and LUS instead of chest CT to detect and define the severity of ARDS.

### Antiviral treatment before ICU admission.

Thailand has set national guidelines for antiviral treatment. Patients with mild symptoms receive chloroquine or hydroxychloroquine plus a boosted protease inhibitor, lopinavir or darunavir plus ritonavir. Favipiravir is not recommended in mild cases because of its limited availability.^8^

Favipiravir is an antiviral RNA polymerase inhibitor for which most preclinical data are derived from its influenza and Ebola activity.^16^ It is given to all patients with proven COVID-19 who have symptoms or signs consistent with pneumonia, or when there is hypoxemia (SpO_2_ < 95% on room air).^8^

## INTENSIVE CARE UNIT MANAGEMENT

In Thailand, ICU management of COVID-19 is limited to affordable measures (Table 2).

**Table 2:** Critical care management in COVID-19 in Thailand

Measure	Purpose/aim
Infection prevention and control in intensive care unit	
* *Increasing availability of personal protective equipment	Protection of frontline health care workers and other patients
* *Free up IARRs	
* *Minimizing aerosol-generating procedures	
* *Reuse of decontaminated disposable filtering facepiece respiratorss	
Respiratory support	
Supplemental oxygen	
* *Use lowest possible fraction of inspired oxygen	Avoid oxygen toxicity
* *Permissive hypoxia (SpO_2_ of 88% or higher)	Avoid oxygen toxicity
* *Consider nasal prong in mild dyspnea	Cheap resource
* *Consider a non-rebreathing mask in moderate dyspnea	Prevention of intubation
* *Consider high-flow nasal oxygen/noninvasive ventilation in severe dyspnea (needs availability of airborne infection isolation room)	Prevention of intubation
Prone positioning	
* *Low threshold for prone or recovery positioning in awake patients	Prevention of intubation
Invasive ventilation	
* *Use low tidal volumes, that is, 6–8 mL/kg predicted body weight	Lung protection
* *Permissive hypercarbia (blood pH of 7.2 or higher) with permissive hypoxia (SpO_2_ of 88% or higher or PaO_2_ of 8 kPa or higher)	
* *Caution when PEEP higher than 10 cm H_2_O	Avoiding overdistension
* *Only use higher PEEP when it results in a lower driving pressure	
* *Keep driving pressure below 15 cm H2O and plateau pressures of < 30 cm H_2_O	
Fluid management	
* *Restrictive fluid therapy	Avoid pulmonary edema
* *Early use of vasopressors	Maintain organ perfusion pressure and prevent acute kidney injury
* *Use loop diuretic, and renal replacement therapy only if AKI results in a too positive fluid balance	Avoid aggravation of pulmonary edema and worsening hypoxemia

IARRs = Incremental Auction Revenue Rights; SpO_2_ = peripheral capillary oxygen saturation; PEEP = positive end-expiratory pressure.

### Infection control in the ICU.

Availability and proper use of isolation and personal protective equipment (PPE) are essential to protect frontline healthcare workers, as well as other patients without COVID-19. As in other countries, Thailand has a shortage of negative pressure rooms and PPE. Only severe cases or cases that undergo aerosol-generating procedures can be placed within negative pressure rooms, if available. Aerosol-generating procedures, such as collection of respiratory specimens, bronchoscopy, and cardiopulmonary resuscitation, should be minimized or avoided. Metered dose inhalers are used for inhaled medications, including for intubated patients. Disposable filtering facepiece respirators (FFRs) are not approved for routine decontamination and reuse as standard of care. However, FFR decontamination and reuse is considered a crisis capacity strategy*.*

### Respiratory support.

**Figure 1.**
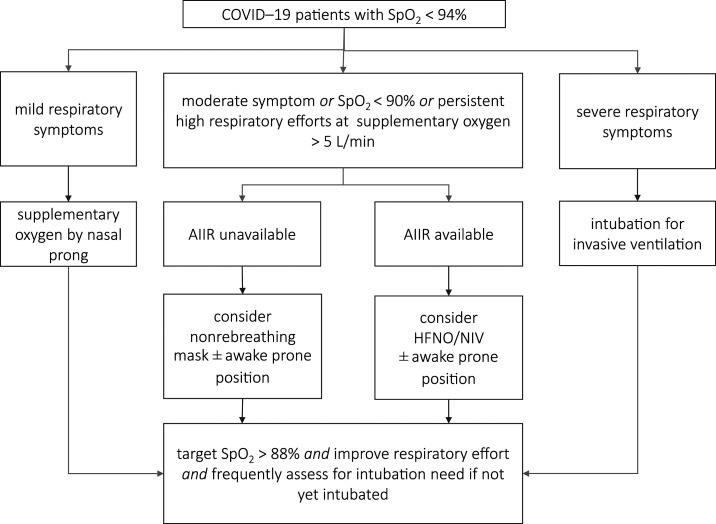
Respiratory support in COVID-19 patients; flowchart of the protocol used in Siriraj Hospital, Mahidol University, Bangkok, Thailand. AIIR = airborne infection isolation room; NIV = noninvasive ventilation; SpO_2_ = peripheral oxygen saturation.

Supplementary oxygen is a first step in respiratory support of COVID-19 patients with hypoxemia. Oxygen is provided either by nasal prongs or a non-rebreather mask. The use of high-flow nasal oxygen (HFNO) and noninvasive ventilation (NIV) is not supported when airborne infection isolation rooms (AIIRs) are not available because of infection control concerns. In case an AIIR is available, HFNO can be used in younger patients without comorbidities who do not tolerate nasal cannula, or with NIV with a dual limb system in morbid obesity or COPD patients (Figure 1). Their use is weighed against the assumption that these therapies often fail to prevent the need for invasive ventilation in COVID-19 patients.

In our experience, hypoxemia is often remarkably well tolerated with COVID-19 (“happy hypoxia”), in particular in younger patients. This is notably different from the case with other causes of severe pneumonia and ARDS,^17^ and hypoxemia alone, even if severe, is not seen as a valid reason to intubate a patient for invasive ventilation.

Awake COVID-19 patients requiring > 2 L/minute of oxygen to maintain SpO_2_ > 94% or with a PaO_2_/FiO_2_ < 200 mmHg are considered candidates for awake prone positioning when there is no contraindication.^18^ This approach may improve oxygenation without the need for additional resources and is of immense value during a surge of COVID-19 patients, especially in resource-limited settings. However, because awake prone positioning may not be tolerated for a prolonged period of time, we frequently use a lateral recumbent or three-quarters prone (recovery) position^19^ for 2–4 hours, alternating with prone positioning.

Intubation is not to be delayed until the patient acutely decompensates, with spontaneous vigorous inspiratory effort that may cause self-induced lung injury.^20^ We have a low threshold to intubate those who fail to improve or rapidly progress over a few hours despite oxygen supplementation, or who develop hypercapnia, hemodynamic instability, or multi-organ failure (Figure 1).

Invasive ventilation in patients with critical COVID-19 differs in several aspects from that in patients with ARDS from other causes. One important difference is the coexistence in COVID-19 of severely affected noncompliant lung tissue adjacent to relatively compliant unaffected areas. Whereas affected areas cannot be opened, or are very difficult to open with recruitment maneuvers and higher positive end-expiratory pressure (PEEP), unaffected areas are at risk of overdistension by such pressure levels. In these patients, strategies to prevent atelectrauma with high levels of PEEP could in fact be harmful.^21^ However, this may not be true for all patients. It has been suggested that a subset of patients may have recruitable COVID-19 lesions that respond to higher PEEP.^22,23^ This may be evaluated with a chest CT scan at two different PEEP levels, although this approach is difficult in settings where resources are limited. Our initial response to intubated patients with COVID-19 is to set PEEP at ∼10 cm H_2_O and to do a trial with higher PEEP only in cases with a high driving pressure. Ventilation is continued with higher PEEP only when this results in a drop in the driving pressure.

### Fluid management.

**Figure 2.**
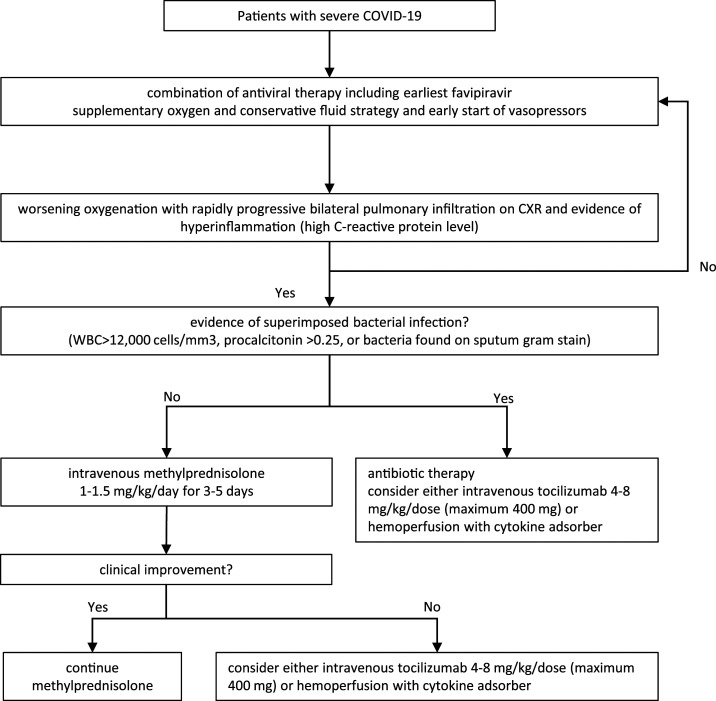
Critical care management in critically ill COVID-19 patients; flowchart of the protocol used in Siriraj Hospital, Mahidol University, Bangkok, Thailand.

A restrictive fluid strategy may be important to avoid aggravation of pulmonary edema. In our patients, if organ perfusion is appropriate, fluid boluses are avoided. It has been suggested that some patients develop the so-called cytokine storm or hyperinflammatory phase, in which hypotension and hypoperfusion may respond well to fluid administration. We continue a restricted fluid approach also in cases of hypotension (Figure 2). Early application of vasopressors concurrent with proper fluid resuscitation is used to provide good tissue perfusion with limited fluid therapy. Loop diuretics are recommended in case the fluid balance becomes too positive, and renal replacement therapy may be necessary to correct the fluid balance in some patients.

### Secondary infections.

COVID-19 patients frequently need prolonged intubations and stays in the ICU, and thus may develop typical nosocomial complications such as ventilator-associated pneumonia and sepsis. In this setting, these infectious complications are diagnosed and treated as for any hospitalized patient.

## ADJUNCTIVE THERAPIES

Clinical trials evaluating the effects of favipiravir, hydroxychloroquine, chloroquine, lopinavir or darunavir plus ritonavir, remdesivir, and several other compounds in COVID-19 are planned or currently underway.^16^ Currently, there is no evidence from randomized clinical trials that any of these adjunctive therapies improve outcomes in patients with severe COVID-19. Several of these therapies have been included in the Thai national guidelines,^8^ albeit at a limited level (Tables 3 and 4). Available evidence may be insufficient to make a recommendation, as is a usual practice in high-income countries.

**Table 3:** Adjunctive therapies for use in COVID-19 in Thailand

Measure	Purpose/aim
* *Antiviral strategies	
* *Favipiravir + chloroquine or hydroxychloroquine + darunavir/ritonavir or lopinavir/ritonavir	May have therapeutic effects
* *Close monitoring for side effects	To prevent harm by side effects
Immunomodulatory therapies	
Corticosteroids	
* *Avoid routine use of corticosteroids	Prevention of secondary infections
* *Consider methylprednisolone 1.0–1.5 mg/kg/day for 3–5 consecutive days	Prevent fibrosis
Tocilizumab	
* *Consider tocilizumab 4–8 mg/kg/dose (maximum 400 mg)	May mitigate the inflammatory response
Cytokine adsorption therapy	
* *Consider hemoperfusion for 3–5 days	May mitigate the inflammatory response
Anticoagulant therapy	
Always give prophylactic LMWH	Prophylaxis against thromboembolism
Consider therapeutic LMWH	Treatment of peripheral thrombosis or pulmonary embolism

LMWH = low molecular weight heparin.

**Table 4:** Patient characteristics and provided treatments in COVID-19 patients admitted to the intensive care unit at Siriraj Hospital, Bangkok, Thailand

	*N* = 13*
Age (years), mean ± SD	58 ± 15
Gender	8 (62)
Male, *n* (%)	
Comorbidities	
Hypertension, *n* (%)	7 (54)
Diabetes mellitus, *n* (%)	7 (54)
Body mass index (kg/m^2^), mean ± SD	29.3 ± 7.0
Partial pressure of oxygen/fraction of inspired oxygen on admission (mmHg)	171 ± 97
C-reactive protein (mg/L)	136 ± 93
Respiratory support, *n* (%)	
High-flow nasal oxygen	11 (85)
Noninvasive ventilation	4 (31)
Invasive ventilation	5 (38)
Prone positioning	9 (69.23)
While Awake	5 (38)
While intubated	4 (31)
Course of methylprednisolone, *n* (%)	6 (46)
Course of interleukin 6 inhibitor (tocilizumab), *n* (%)	3 (23)
Hemoperfusion with cytokine absorber, *n* (%)	3 (23)
Intensive care unit length of stay (days)	17 ± 9
Mortality at the longest follow-up, *n* (%)	0 (0)

* All patients received combined antiviral medications as clinical practice guideline for COVID-19 in Thailand, and none required extracorporeal membrane oxygenation.

### Antiviral and antimalarial strategies.

In Thailand, awaiting definitive results of randomized clinical trials, critically ill COVID-19 patients receive combination therapies with at least three different mechanisms of action, including favipiravir for 10 days, depending on clinical symptoms. Because of availability and affordability, the combination typically includes favipiravir, chloroquine or hydroxychloroquine, and darunavir/ritonavir or lopinavir/ritonavir. Adding azithromycin is optional.^24^

Patients are closely monitored for side effects such as diarrhea, nausea, and hepatitis, as well as potential drug interactions. Patients who receive darunavir/ritonavir or lopinavir/ritonavir in combination with azithromycin for more than 5 days are monitored by daily electrocardiogram (ECG). If the ECG reveals a QTc > 480 milliseconds, discontinuation of darunavir/ritonavir, lopinavir/ritonavir, or azithromycin should be considered. Because favipiravir may be teratogenic, this agent should not be given to women in the reproductive age.^8^

### Immunomodulatory therapies.

Immunomodulatory therapies with promising results are corticosteroids,^25^ a monoclonal antibody interleukin 6 (IL-6) receptor antagonist,^26^ immunoglobulin therapy,^27,28^ and cytokine adsorption therapy.^29^ The rationale for their use is that the underlying pathophysiology of significant organ damage in the lungs and other organs is caused by a cytokine storm.^30^

The potential benefit of corticosteroid therapy^16,31^ may be outweighed by its adverse effects, including delayed viral clearance^32^ and an increased risk of secondary infections. Despite the finding that administration of methylprednisolone was associated with a decreased risk of death in one retrospective study of COVID-19 patients in China,^25^ in Thailand, routine use of corticosteroids is not recommended.^33^ In cases with progressive opacities on CXR where bacterial infection is considered unlikely, we consider methylprednisolone for 3–5 days (Figure 2). It is to be used with caution and stopped immediately in case of suspected or confirmed bacterial superinfection.

Tocilizumab (TCZ) is a monoclonal antibody against the IL-6 receptor that possibly mediates SARS-CoV-2–associated inflammation. Tocilizumab has been used to treat rheumatoid arthritis and approved by the FDA for treating cytokine release syndrome.^34^ In one retrospective study in China, in COVID-19 patients with respiratory distress, hypoxemia, or requiring ICU support, a single dose of TCZ was associated with clinical improvement.^35^ Because of costs and limited availability, TCZ is only occasionally used in Thailand.

Blood purification therapy may have beneficial effects.^29^ In Thailand, hemoperfusion with cytokine adsorbent is sporadically used in patients who were unresponsive to or had contraindication for corticosteroids (Figure 2).

### Anticoagulant therapy.

With emerging evidence that peripheral thrombosis and pulmonary embolism are very common in COVID-19 patients and that microthrombi may be responsible for much of the pathophysiology,^36^ we strongly favor the use of low molecular weight heparin as a prophylactic therapy. In case of confirmed peripheral thrombosis and pulmonary embolism, aggressive anticoagulant therapy should be started immediately.

## CONCLUSION

The COVID-19 pandemic represents the greatest global public health crisis of our generation. To date, no specific therapies have been shown effective. Although public health policies aimed at preventing new COVID-19 cases, including assistance from the general public, are far more important than advanced medical technologies, governments, hospital administrators, and policy-makers must collaborate with ICU practitioners to tackle the challenges of ICU care for COVID-19. The approach presented here may serve as an example for other countries and regions facing similar restrictions in care.
